# Bilateral Trade Flows and Income Distribution Similarity

**DOI:** 10.1371/journal.pone.0128191

**Published:** 2016-05-03

**Authors:** Inmaculada Martínez-Zarzoso, Sebastian Vollmer

**Affiliations:** 1 Department of Economics, University Jaume I, Castellón de la Plana, Spain; 2 Department of Economics, University of Göttingen, Göttingen, Germany; University of Florida, UNITED STATES

## Abstract

Current models of bilateral trade neglect the effects of income distribution. This paper addresses the issue by accounting for non-homothetic consumer preferences and hence investigating the role of income distribution in the context of the gravity model of trade. A theoretically justified gravity model is estimated for disaggregated trade data (Dollar volume is used as dependent variable) using a sample of 104 exporters and 108 importers for 1980–2003 to achieve two main goals. We define and calculate new measures of income distribution similarity and empirically confirm that greater similarity of income distribution between countries implies more trade. Using distribution-based measures as a proxy for demand similarities in gravity models, we find consistent and robust support for the hypothesis that countries with more similar income-distributions trade more with each other. The hypothesis is also confirmed at disaggregated level for differentiated product categories.

## 1. Introduction

The role of within-country income distributions and between-country income distribution similarities as explanatory factors of the pattern of trade across countries has been a relatively neglected area in international trade with respect to other factors, namely total incomes and differences in factor endowments. Most trade theories, including Ricardian models [1], neoclassical models [2] and new trade theories [3, 4], assume that preferences are homothetic and identical across countries, giving none or a very small role to demand patterns as factors that can explain the volume of international trade flows (to which we refer through the paper as volume in monetary units, namely US-Dollar). This assumption might have been useful to simplify the modeling framework, but it was based on a weak empirical foundation. A number of studies clearly find that consumer preferences are non-homothetic [5, 6, 7]. For instance, non-homothetic tastes imply that the ratios of goods demanded by consumers not only depend on relative prices, as it is the case under the usual homothetic-preference assumption, but also on their income.

An early exception to the main strand of theoretical models is the well-known Linder hypothesis [8]. Linder departs from traditional trade theory where supply side factors, namely differences in technologies and factor endowments between countries, are the main determinants of the volume of trade. He argued that the traditional theories cannot explain why countries would engage in both exports and imports of the same type of products. Linder considers that demand for a product has to appear first in the producer country and then this product can be exported to other countries that have similar demand structures.

Recently, Fajgelbaum et al. [9], Fieler [10] and Markusen [11] incorporated the assumption of non-homothetic consumer preferences in general equilibrium models of international trade. The theoretical model of Fajgelbaum et al. [9] predicts that richer countries will be net exporters of higher quality goods and net importers of lower quality goods under reasonable assumptions about levels and distribution of national income. The model also implies that in most cases trade liberalization benefits the poorer households in wealthy countries and the richer households in poor countries. Fieler [10] proposes a model that explains both North–North (among rich countries) and North–South (among rich and poor countries) patterns. The volume of trade among rich countries occurs primarily in differentiated goods, while trade of rich with poor countries occurs across sectors. Finally, Markusen [11] builds a generic model of identical but non-homothetic preferences and presents a unified and testable set of results. Among them, he predicts higher mark-ups and higher price levels in high income countries (high productivity economies) and that for two countries with the same average income, aggregate demand for the luxury will be higher in the country with the more unequal distribution.

With respect to the related empirical literature, we find several studies that test the Linder hypothesis. Early studies are summarized in McPherson, Redfearn and Tieslau [12, 13]. In most cases a gravity model was used extended with income similarity variables. The gravity model, first introduced by Tinbergen [14] and Pöyhönen [15] in the international trade literature has been widely used as an empirical tool to analyse the determinants of bilateral trade flows as it provides a good fit to most data sets of international trade flows. Bilateral trade is defined as trade between pair of countries at the sectorial level (volume in US Dollars). In our empirical estimation we specifically state whether the within product or across product variation of bilateral trade is explained.

In a generalized gravity model, trade between two countries is positively related to the size of the economies and negatively related to the distance, a proxy for transportation costs, between them. In addition, a number of bilateral factors that foster or impede trade are usually included as explanatory variables. Differences in income per capita is the variable selected to measure income similarities between trading pairs in most papers [16, 17]. More recent studies include Choi [18], Martínez-Zarzoso and Nowak-Lehmann [19] and Hallak [20]: the latter two use disaggregated trade flows. Hallak [20] focuses on product quality and shows that the failure to confirm the Linder hypothesis in past studies could be due to aggregation bias. He finds support for the Linder hypothesis by testing it for different types of products separately.

Most of the above mentioned studies consider per capita income differences between countries. A few recent studies also consider the within country distribution of income as a determinant of bilateral trade flows: Hunter [5], Francois and Kaplan [21], Matsuyama [22] and Mitra and Trindade [23], Bohman and Nilsson [24], Choi et al. [25] are some of them. We aim at integrating both approaches.

First, we aim at evaluating the effect of within country income inequality on the volume of international trade flows at a disaggregate level. Second, we estimate the effect of income distribution similarities on bilateral trade, controlling for within country income inequalities (Gini coefficient) and for differences in income per capita between countries (Linder term), as well as controlling for time-invariant factors that are specific to each country pair, as for example cultural differences. To accomplish our second goal, we provide new measures for the similarity of demand structures between countries based on similarity of within country income distribution. To construct the index, we first estimate the distribution of income within each country and then we measure to what extent the distributions of two given countries overlap. The underlying assumption is that the overlap between the respective density functions of income within each country can be considered as a good proxy for the similarity in the demand structure between trading partners. This assumption has been made by Fajgelbaum et al. [9], and justified by the fact that demand differences are not caused by exogenous variations in tastes across countries, but rather derive from differences in income distribution. Also empirically, a robust relationship has been found between per capita income and the composition of national consumption [26, 27]. The proposed measure of demand similarity is added as explanatory variable in a gravity model of trade that is also augmented with within country inequality measures and with per capita income differences. The main advantage of the density functions used in this paper with respect to Choi et al.’s [25] measure is that we are able to obtain full density functions for more than a hundred countries and for different periods, whereas Choi et al. [25] restricted their analysis to 26 countries and constrained their analysis to a single wave of income data.

The results from estimating the theoretically justified gravity model of trade show a positive effect of within country inequality in the destination country on bilateral trade, and a significant and economically important effect of similarity of demand structures (measured by the overlap of income distributions) on bilateral disaggregated trade flows.

In the next Section, we explain how to construct the measure for income distribution similarity. In Section 3, we conduct our empirical analysis and present the main results before concluding in Section 4.

## 2. Income Distribution Overlaps between Countries

We assume that national income distributions follow a log-normal distribution. Formally, the log-normal distribution LN(μ,σ) is defined as the distribution of the random variable Y = exp(X), where X has a normal distribution with mean µ and standard deviation σ. It can be shown that the density of LN(μ,σ) is,

f(x;μ,σ)=1xσ2πe−(log(x)−μ)22σ2. (1)

The Gini coefficient G of LN (µ,σ) is given by G = 2Φ(σ/√2) − 1, where Φ is the distribution function of the standard normal distribution. Therefore, the parameters µ and σ of LN (µ,σ) can be determined from the average income E(Y) and the Gini coefficient G as follows.

$$\sigma =\sqrt{2\phi^{-1}}\;\left(\frac{\text{G}+1}{2}\right),\;\mu =\log\left(\text{E}\left(\text{Y}\right)\right)-\sigma^{2}/2.$$ (2)

The log-normal distribution is only a very rough approximation of national income distributions. With a large enough micro data set, one could most likely reject the log-normal assumption–as one could reject any other simple parametric assumption. For the available macro data however, the log-normal assumption turns out to be a quite good approximation for national income distributions. Lopez and Serven [28] test the log-normal assumption systematically for a large number of countries and years for which both the Gini coefficient and quintile income shares are available (about 800 country-year observations). They find that log-normality cannot be rejected for income data.

Income data are drawn from the Penn World Tables 6.2 [29], which report the real GDP per capita in constant international dollars (chain series, base year 2000), available for most countries. For three particularly populous countries, namely Bangladesh, Russia and Ukraine we estimated the initial missing values. For Bangladesh we calculated the values for the two initial years 1970, 1971 using the average income per capita growth rate of the rest of the decade. For Russia and Ukraine we used derived USSR growth rates to estimate the average income for the years before 1990. Our second data source is the World Income Inequality Database from UNU-WIDER with the adjustments of Grün and Klasen [30]. The adjusted Gini dataset of Grün and Klasen is derived by using several estimation techniques and has substantial advantages in terms of comparability to the raw Ginis available in the WIDER database, which are not fully comparable over time and across countries. We assume the first real observations of the Gini in any given country to be equal to its initial level of inequality. Starting from this initial level we used a moving average to capture changes in trends of inequality. Unfortunately, there is no reliable inequality data for the populous Democratic Republic of Congo, hence we used the neighboring Central African Republic’s Gini as a substitute.

Let f_*i*_(x;*μ*_*i*_,*σ*_*i*_) denote the log-normal income density of country i and let f_*j*_(x;*μ*_*j*_,*σ*_*j*_) denote the corresponding income density of country j. Let d_1_,d_2_ ≥ 0 denote the income values at which the two density functions intersect. In practice, for our data, the second intersection happens at income levels at which the density function already approaches the x-axis. We thus assume that each pair of income density functions has one unique income level d ≥ 0 at which the density functions intersect. This assumption simplifies the presentation in this section tremendously and does not have any negative consequences for the precision of our similarity measures. Without loss of generality, we assume that the average income is lower or equal in country i than in country j.

Three measures for the similarity of income distributions of two countries *i* and *j* are proposed. We define *S1*_*ij*_ as the area overlap of the two density functions f_*i*_(x;*μ*_*i*_,*σ*_*i*_) and f_*j*_(x;*μ*_*j*_,*σ*_*j*_). *S1*_*ij*_ can be calculated as follows:

$$S1_{ij}=\int_{0}^{\infty }\text{min}\{f_{j}\left(x;\mu_{j},\sigma_{j}\right),f_{i}\left(x;\mu_{i},\sigma_{i}\right)\}dx$$ (3)

By definition, each density function has an area equal to one. Thus, the overlap *S1*_*ij*_ is a number between zero (no overlap) and one (identical density functions). *S1*_*ij*_ is symmetric and it represents the overall similarity (overlap) of the two income distributions. We interpret *S1*_*ij*_ as a measure for the similarity of the demand structure in countries *i* and *j*.

However, not only the overall similarity of the demand structure is of importance for the volume of trade, but also the number of potential customers. Hence, we propose two additional measures of demand similarity that take population size into account. Let p_*i*_,p_*j*_ denote the population sizes of countries i and j. We define *S2*_*ij*_ as the number of people in country *i* that have a match in country *j*, that is, a person in country *j* with equal income. To this end, we multiply each country’s income density function by its respective population size. *S2*_*ij*_ can be calculated as follows:

$$S2_{ij}=\int_{0}^{\infty }\text{min}\{p_{j}f_{j}\left(x;\mu_{j},\sigma_{j}\right),p_{i}f_{i}\left(x;\mu_{i},\sigma_{i}\right)\}dx$$ (4)

*S2*_*ij*_ is also symmetric. It is a combined measure of similarity of the demand structure and market size. Our third measure, *S3*_*ij*_, is the percentage of country *i*’s population that has a match in country j in terms of income. It is defined as follows

$$S3_{ij}=S2_{ij}/p_{i}$$ (5)

**Fig 1:**
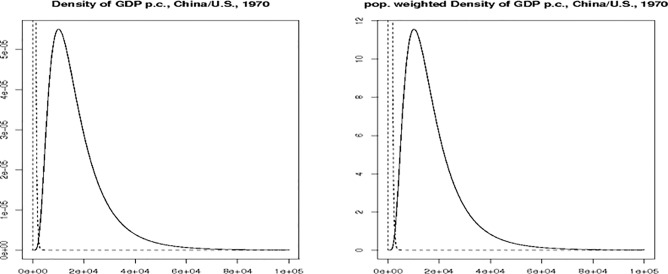
Illustration of Overlaps for China and the U.S., 1970. (A) Left figure: Density of GDP p.c. for China (dashed line) and the U.S. (solid line). (B) Right figure: Density of GDP p.c. For China (dashed line) and the U.S. (solid line) multiplied by population size.

**Fig 2:**
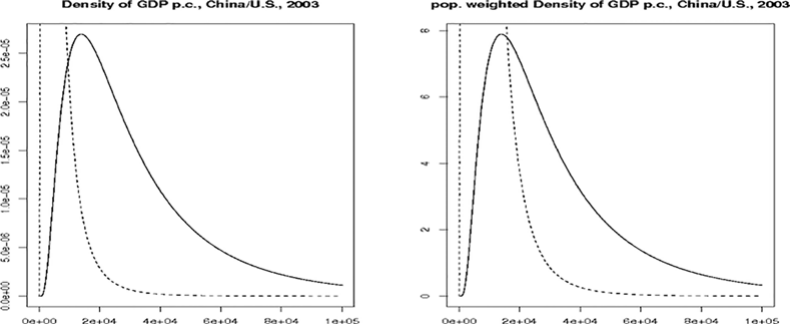
Illustration of Overlaps for China and the U.S., 2003. (A) Left figure: Density of GDP p.c. for China (dashed line) and the U.S. (solid line). (B) Right figure: Density of GDP p.c. For China (dashed line) and the U.S. (solid line) multiplied by population size.

Figs 1 and 2 illustrate *S1*_*ij*_, *S2*_*ij*_ and *S3*_*ij*_ for China and the U.S. in 1970 and 2003. Note that the figures focus on the part of the plot where the two density functions overlap; we have cut out an important part of China’s distribution for a better visibility of the overlap. In 1970, both the overlap (Fig 1) and the population weighted overlap (Fig 2) of the two densities are virtually zero, for about 825,000 people a match can be found in the other country’s population. Most of the mass of the U.S. density is right of the Chinese density: this means that the top percentile in the Chinese income distribution in 1970 was approximately as well off as the bottom percentile in the United States.

This picture changes over time as both the simple area overlap and the population-weighted area overlap increase substantially. In 2003, the area overlap of the two density functions is 22 percent. More than one hundred million people have a match in the other country in terms of income. In other words, 10 percent of the Chinese population and 44 percent of the U.S. population have a match in the other country’s population in terms of income.

## 3. Empirical Evidence

### 3.1 Estimation Strategy

The indices for similarity of income distributions are introduced in a gravity model of trade to evaluate the effect of income distribution similarity on the volume of export between countries. According to the theory, a similar within-income-distribution between countries is expected to have a positive effect on bilateral exports.

According to the generalized gravity model of trade, the volume of sectoral exports between pairs of countries *X*_*ijk*_ is a function of their incomes (GDPs), their incomes per capita, their geographical distance and other trade cost factors as shown by the equation

$$X_{ijk}=\beta_{0}\;Y_{i}^{\beta_{1}}Y_{j}^{\beta_{2}}YH_{i}^{\beta_{3}}YH_{j}^{\beta_{4}}DIST_{ij}^{\beta_{5}}F_{ij}^{\beta_{6}}\;u_{ijk}$$ (6)

where *Y*_*i*_ *(Y*_*j*_*)* indicates the GDPs of the exporter (importer), *YH*_*i*_ *(YH*_*j*_*)* are exporter (importer) GDP per capita, *DIST*_*ij*_ measures the distance between the two countries’ capitals (in the actual estimation it will be absorbed by the fixed effects), and *F*_*ij*_ represents any other factors aiding or preventing trade between pairs of countries. *u*_*ijk*_ is the error term. We augment the gravity equation with Gini coefficients for each country (*Gini_it*, *Gini_jt*) to account for within country inequalities. Further, we augment the gravity equation with each of the income-distribution indices derived in the previous section (*S1*_*ijt*_, *S2*_*ijt*_ and *S3*_*ijt*_). For estimation purposes, and with a time dimension added, we first specify an augmented version of the gravity model in log-linear form given by:

$$\begin{array}{l} \ln\;X_{ijkt}=\alpha_{0}+\phi_{t1}+\chi_{ijk}+\beta_{11}lnY_{it}+\beta_{21}lnY_{jt}+\beta_{31}lnYH_{it}+\beta_{41}lnYH_{jt}\\ +\beta_{41}SI_{ijt}+\beta_{51}Gini_{it}+\beta_{61}Gini_{jt}+\upsilon_{ijkt} \end{array}$$ (7)

where *ln* denotes variables in natural logs, *X*_*ikjt*_ are product *k* exports from country *i* to country *j* in period *t* at current US$. Note that *SI* variables vary over *i*, *j* and *t*, whereas the Gini coefficients are specific for each country and year. *Y*_*it*_, *Y*_*jt*_ indicate the GDP of countries *i* and *j* respectively, in period *t* at constant PPP US$. *YH*_*i*_ and *YH*_*jt*_ denote the income per capita of countries *i* and *j* respectively, in period *t* at constant PPP US$ per thousand inhabitants.

*ϕ*_*t*_ are time effects that control for omitted variables that are common for all trade flows and vary over time. *χ*_*ijk*_ are exporter-importer-industry effects that control for time invariant unobserved heterogeneity that is specific to each industry (3-digit level) and trade flow. *υ*_*ijkt*_ denotes the error term.

Next, we consider country-time effects to account for time-variant multilateral price terms, as proposed by Baldwin and Taglioni [31] and Baier and Bergstrand [32]. As stated by Baldwin and Taglioni [31], including time-varying country dummies should completely eliminate the bias stemming from the “gold-medal error” (the incorrect specification or omission of the terms that Anderson and van Wincoop [33] called *multilateral trade resistance*). This approach involves a large number of dummy variables. However, we have enough degrees of freedom available. A shortcoming of this specification is that we cannot estimate the coefficients of GDP, GDP per capita and Gini indices because they are country specific and vary over time but not bilaterally.

The specification that accounts for the multilateral price terms in a panel data framework is given by

$$\ln\;X_{ijkt}=\alpha_{ijk}-\ln\;P_{it}^{1-\sigma }-\ln\;P_{jt}^{1-\sigma }+\gamma_{1}SI_{ijt}+\varepsilon_{ijkt}$$ (8)

where $P_{it}^{1-\sigma }$and $P_{jt}^{1-\sigma }$are time-varying multilateral (price) resistance terms that will be proxied with *time-varying* country dummies and *ε*_*ijkt*_ denotes the error term that is assumed to be well behaved. The other variables are the same as in Eq 7, above.

### 3.2 Data and results

Different versions of the models specified in the previous section are estimated for disaggregated exports (ISIC 3-digits) using a sample of 104 exporter and 108 importers for which income distribution data are available [34, 35, 36]. The period under study is from 1980 to 2003 and we are considering data for 1980, 1985, 1990, 1995, 2000 and 2003. The descriptive statistics presented in Table 1 indicate that income overlap patterns include valuable information that average values (differences in income per capita) are not able to capture.

**Table 1:** Development of income similarity indices over time (example China and the U.S.).

Year	S1	S2	S3 CHN	S3 USA
1970	.002	825	.001	.004
1975	.004	1462	.002	.007
1980	.008	3574	.004	.015
1985	.023	9599	.009	.039
1990	.054	26079	.023	.102
1995	.114	58117	.048	.216
2000	.165	88347	.070	.311
2003	.221	128216	.100	.438

Note: SI 1 and 3 are index values (range 0 to 1). SI 2 is measured in thousands of people.

Table 2 presents summary statistics of the main variables used in the analysis. Our main focus is on income per capita, within country income inequality and between country income-similarity variables (Indices S1, S2 and S3 described above).

**Table 2:** Summary statistics.

Variable	Obs.	Mean	Std. Dev.	Min	Max
**Log of bilateral exports**	481766	5.852	3.176	-0.691	18.014
**S1**	645960	0.448	0.291	0.001	0.998
**S2**	645960	8.309	1.373	1.569	17.963
**S3**	645960	0.402	0.359	0.001	1.000
**Log GDP of exporter countries**	645960	25.86	1.690	20.404	29.954
**Log GDP of importer countries**	645960	25.54	1.809	19.808	29.954
**Log GDP p.c. of exporter countries**	645960	9.013	1.035	6.186	10.459
**Log GDP p.c. of importer countries**	645960	8.806	1.109	5.884	10.459
**Gini of exporter countries**	645960	0.433	0.091	0.238	0.7920
**Gini of importer countries**	645960	0.444	0.097	0.238	0.7920

Note: Log indicates natural logarithms. S1, S2 and S3 are measures of income distribution similarities as described in Section 2: S1 is a measure for similarities in the demand structure (Eq 3). S2 is a measure for similarities in demand structure and market size (Eq 4). S3 is a measure for population overlaps in terms of income (Eq 5).

Table 3 presents the estimation results for Eq (7) with exporter-importer-industry and year fixed effects and with robust standard errors clustered across industries. The first column shows the effect of income per capita differences on the volume of trade. The estimated coefficient is negative and statistically significant.

**Table 3:** Income similarity, inequality and exports.

		S1	S2	S3	Income p.c. difference
	(1)	(2)	(3)	(4)	(5)
**Income p.c. difference**	-0.248***		-0.183***	-0.213***	-0.251***
	(0.022)		(0.024)	(0.024)	(0.022)
**Similarity index**		0.000	0.114***	0.242***	
		(0.060)	(0.017)	(0.071)	
**Gini of exporter countries**		-0.300	-0.452***	-0.294***	-0.257**
		(0.200)	(0.113)	(0.109)	(0.108)
**Gini of importer countries**		0.996***	0.909***	0.960***	1.019***
		(0.126)	(0.086)	(0.086)	(0.085)
**Log GDP of exporter countries**	2.119***	2.052***	2.045***	2.141***	2.106***
	(0.053)	(0.216)	(0.054)	(0.054)	(0.053)
**Log GDP of importercountries**	-0.688***	-0.717***	-0.713***	-0.673***	-0.638***
	(0.044)	(0.163)	(0.045)	(0.045)	(0.044)
**Log GDP p.c. of exporter countries**	-0.590***	-0.520**	-0.508***	-0.602***	-0.572***
	(0.058)	(0.193)	(0.059)	(0.059)	(0.058)
**Log GDP p.c. of importer countries**	1.857***	2.060***	1.855***	1.827***	1.806***
	(0.046)	(0.168)	(0.047)	(0.047)	(0.046)
**Dummy for 1985**	-0.223***	-0.234***	-0.222***	-0.225***	-0.226***
	(0.01)	(0.027)	(0.01)	(0.01)	(0.01)
**Dummy for 1990**	0.184***	0.146***	0.175***	0.169***	0.169***
	(0.014)	(0.048)	(0.014)	(0.014)	(0.014)
**Dummy for 1995**	0.465***	0.412***	0.449***	0.440***	0.441***
	(0.019)	(0.07)	(0.019)	(0.019)	(0.019)
**Dummy for 2000**	0.232***	0.161	0.220***	0.206***	0.207***
	(0.025)	(0.097)	(0.025)	(0.025)	(0.025)
**Dummy for 2003**	0.283***	0.211*	0.272***	0.257***	0.257***
	(0.028)	(0.113)	(0.028)	(0.028)	(0.028)
**Constant**	-43.069***	-43.581***	-42.479***	-44.087***	-44.054***
	(1.238)	(3.882)	(1.265)	(1.238)	(1.238)
**R-Squared**	0.223	0.223	0.224	0.224	0.224
**N**	481766	481766	481766	481766	481766
**LL**	-757206	-757245	-757007	-757038	-757052
**RMSE**	1.165094	1.165191	1.164618	1.164692	1.164726

Note: Log indicates natural logarithms. S1, S2 and S3 are measures of income distribution similarities as described in Section 2: S1 is a measure for similarities in the demand structure (Eq 3). S2 is a measure for similarities in demand structure and market size (Eq 4). S3 is a measure for population overlaps in terms of income (Eq 5). Income p.c. difference is included as absolute value. The similarity index denotes S1 in column (2), S2 in column (3) and S3 in column (4). Gini denotes the Gini inequality index. Robust standard errors clustered by country pair are reported below each estimate. Exporter-importer-industry and time fixed effects are included, the first set of fixed effect is not reported to save space.

*, **, *** denote statistically significance at the 10, 5 and 1 percent level, respectively.

Columns 2 to 4 show the effect of the similarity indices S1, S2 and S3, respectively. Their effect on the volume of trade is positive and statistically significant for indices S2 and S3 and statistically insignificant for index S1. It is also worth noting that the inclusion of the indices on exports slightly reduces the effect of the traditional Linder term, but both seem to proxy for different effects since they are simultaneously significant.

The last column of Table 3 shows the results of adding income per capita differences jointly with Gini inequality indices without the income-similarity indices as explanatory variables. As already found in previous studies [19, 20], the absolute difference in per capita income is negatively related to exports. The coefficient of the Gini index is negative and significant for the exporter and positive and significant for the importer.

Next, we estimate the gravity model for trade between high-income OECD, mid-income and low-income countries with exporter-time and importer-time dummies (Eq 8). Table 4 only includes estimates for variables that have bilateral variation, which means that the effects of income and income per capita variables are subsumed into the country-and-time fixed effects. The coefficients of the similarity indices S2 (column 2, row 3) and S3 (column 3, row 3) are positive and significant (except S3 for low-income countries). The coefficient of S1 (column 1, row 3) is insignificant for all three groups of countries.

**Table 4:** Result for different country groups with multilateral resistance.

**OECD**	**(1)**	**(2)**	**(3)**
	S1	S2	S3
**Income p.c. difference**	0.109	-0.366**	-0.242*
	(0.16)	(0.15)	(0.12)
**Similarity index**	-0.002	0.421***	0.692***
	(0.28)	(0.06)	(0.12)
**R-squared**	0.389	0.385	0.610
**N**	54245	54245	54245
**Mid-income**	**(1)**	**(2)**	**(3)**
	S1	S2	S3
**Income p.c. difference**	-0.206*	-0.054	-0.137***
	(0.11)	(0.06)	(0.05)
**Similarity index**	-0.276	0.565***	0.211**
	(0.27)	(0.04)	(0.11)
**R-squared**	0.328	0.328	0.196
**N**	70006	70006	70006
**Low income**	**(1)**	**(2)**	**(3)**
	S1	S2	S3
**Income p.c. difference**	-0.532**	0.099	-0.441***
	(0.22)	(0.08)	(0.08)
**Similarity index**	0.005	1.064***	-0.165
	(0.49)	(0.09)	(0.18)
**R-squared**	0.269	0.185	0.282
**N**	17523	17523	17523

Note: S1, S2 and S3 are measures of income distribution similarities as described in Section 2: S1 is a measure for similarities in the demand structure (Eq 3). S2 is a measure for similarities in demand structure and market size (Eq 4). S3 is a measure for population overlaps in terms of income (Eq 5). Income p.c. difference is included as absolute value. The similarity index denotes S1 in column (1), S2 in column (2) and S3 in column (3). Robust standard errors are reported below each estimate. Exporter-importer-industry, exporter-year and importer-year fixed effects are included.

*, **, *** denote statistically significance at the 10, 5 and 1 percent level, respectively. Robust standard errors clustered by country pair are reported below each estimate.

In Table 5 we present a summary of the estimation results for Eq (7) with exporter-importer-product fixed effects at the industry level. Here we only consider the similarity index *S2*. The coefficient is positive and statistically significant in 20 industries (column1, row 1) and insignificant in 8 industries (column3, row 1). The full set of regressions for each industry is shown in Table A.3 in the S1 Appendix.

**Table 5:** Summary of sectorial estimations.

	Sign and significance			Pooled regression
	Positive and significant (5%)	Negative and significant (5%)	Non-Significant	Average Coefficients
	(1)	(2)	(3)	(4)
**S2**	20	0	8	0.114***
**Income p.c. difference**	0	23	5	-0.183***
**Gini of exporter countries**	1	9	8	-0.452***
**Gini of importer countries**	14	0	14	0.909***

Note: Index S2 is described in Section 2 and measures income similarity between pairs of countries. Gini denotes the Gini inequality index. Income p.c. difference is included as absolute value. Robust standard errors clustered across pairs of countries. Exporter-importer-product fixed effects are included.

*, **, *** denote statistically significance at the 10, 5 and 1 percent level, respectively.

Overall, these results confirm Hallak’s [20] prediction that income per capita differences have a negative impact on the volume of bilateral trade at the sectoral level. Moreover, our results indicate that differences in the distribution of income between countries impact intra-sectoral trade. In particular, a decrease in these differences increases the volume of trade, also when controlling for differences in per-capita income in the same regression. This result is in accordance with Francois and Kaplan [21] and Choi et al. [25] whose results emphasize the importance of taking into account higher moments of the income distribution.

Finally, concerning within country inequality, the results indicate that the Gini coefficient is negative and significant; hence, redistribution policies that help reduce the Gini coefficient in the exporting country should in most cases have a positive impact on exports.

Sensitivity checks, namely results obtained in regressions at the sectoral level, and results using difference similarity indices, indicate that our results are robust. The results are also robust to the consideration of the zero flow observations by estimating a Heckman-type model and also a two-part model and to the specification of dynamics. The Heckman model controls for selection into exporting and allows the incorporation of zero trade flows in a first step estimation (in the first step the decision to export is modelled using a probit model). In a second step, the volume of exports is used as dependent variable and the inverse Mills ratio obtained from the first estimation is added as explanatory variable as a control for selection bias. The results indicate that controlling for selection does not affect our main results. The same is the case when using as an alternative a two-part model. Dynamics are specified by adding lagged variables to the model, including lagged exports, and estimating the model using a GMM estimator. With regards to the variable of interest SI2, the long-run estimated coefficient equals 0.23 (= 0.137/(1–0.368–0.05)) that is in line with previous results.

## 4. Conclusions

Trade theory in conjunction with some stylized empirical facts indicates that preferences are non-homothetic; not only the average income but also the distribution of income should influence aggregate demand. Ideally, the full distribution of income should be considered when demand similarities between countries are measured. In this paper we present empirical evidence supporting the hypothesis of non-homothetic preferences. We propose three new measures of income distribution similarity between countries. These measures are used to proxy for demand similarities between pairs of countries across trading partners and over time.

Using distribution-based measures as a proxy for demand similarities in gravity models, we find consistent and robust support for the hypothesis that countries with more similar income-distributions trade more with each other. The hypothesis is also confirmed at disaggregated level for differentiated product categories. The larger the overlap in income distribution between two countries, the greater the extent of trade between the two countries.

## Supporting Information

S1 Appendix (DOCX)
