# Supplementary material for: Bilateral Trade Flows and Income Distribution Similarity
Source: PLoS One. 2016 May 3;11(5):e0128191. doi: 10.1371/journal.pone.0128191 (PMC4854469; doi:10.1371/journal.pone.0128191)
Supplement: S1 Appendix — (DOCX) [file pone.0128191.s001.docx]

Appendix

A.1 Description of industries

| **Sectors** | **isic_rev2** | **isic_Industries** |
| --- | --- | --- |
| 31 | 311 | Food products |
|  | 313 | Beverages |
|  | 314 | Tobacco |
| 32 | 321 | Textiles |
|  | 322 | Wearing apparel except footwear |
|  | 323 | Leather products |
|  | 324 | Footwear except rubber or plastic |
| 33 | 331 | Wood products except furniture |
|  | 332 | Furniture except metal |
| 34 | 341 | Paper and products |
|  | 342 | Printing and publishing |
| 35 | 351 | Industrial chemicals |
|  | 352 | Other chemicals |
|  | 353 | Petroleum refineries |
|  | 354 | Miscellaneous petroleum and coal products |
|  | 355 | Rubber products |
|  | 356 | Plastic products |
| 36 | 361 | Pottery china earthenware |
|  | 362 | Glass and products |
|  | 369 | Other non-metallic mineral products |
| 37 | 371 | Iron and steel |
|  | 372 | Non-ferrous metals |
| 38 | 381 | Fabricated metal products |
|  | 382 | Machinery except electrical |
|  | 383 | Machinery electric |
|  | 384 | Transport equipment |
|  | 385 | Professional and scientific equipment |
| 39 | 390 | Other manufactured products |

Source: Trade and Production Dataset. World Bank.

A.2 List of countries

| Algeria | Iraq* | Sudan |
| --- | --- | --- |
| Argentina | Ireland | Suriname |
| Australia | Israel | Swaziland |
| Austria | Italy | Sweden |
| Bahamas | Jamaica | Switz. |
| Bangladesh | Japan | Taiwan |
| Bolivia | Jordan | Tanzania |
| Botswana | Kenya | Thailand |
| Brazil | Korea Rep. | Trinidad Tbg |
| Burkina Faso | Lao P. Dem. R* | Tunisia |
| Burundi | Madagascar | Turkey |
| Cambodia | Malawi | Uganda |
| Cameroon | Malaysia | Ukraine |
| Canada | Mali | Untd. Kingdom |
| Cent.Afr.Rep | Mauritania | Uruguay |
| Chad | Mauritius | USA |
| Chile | Mexico | Venezuela |
| China | Mongolia | Zaire* |
| Colombia | Morocco | Zambia |
| Congo | Mozambique | Zimbabwe |
| Costa Rica | Namibia |  |
| Cote d’Ivoire | Nepal |  |
| Cuba | Netherlands |  |
| Denmark | New Zealand |  |
| Dominican R. | Nicaragua |  |
| Ecuador | Niger |  |
| Egypt | Nigeria |  |
| El Salvador | Norway |  |
| Ethiopia | Pakistan |  |
| Fiji | Panama |  |
| Finland | Papua N..Guinea |  |
| France | Paraguay |  |
| Gambia | Peru |  |
| Ghana | Philippines |  |
| Greece | Poland |  |
| Guatemala | Portugal |  |
| Guinea | Romania |  |
| Guinea Bissau | Russian Fed |  |
| Honduras | S.Afr. |  |
| Hong Kong | Senegal |  |
| Hungary | Sierra Leone* |  |
| India | Singapore |  |
| Indonesia | Spain |  |
| Iran-Islam.R | Sri Lanka |  |

Note: 104 origins and 108 destinations, * indicates available as importer only.

A.3 Sectoral results

|  | **Food** | **Beverages** | **Tobacco** | **Textiles** | **Apparel** | **Leather** | **Footwear** |
| --- | --- | --- | --- | --- | --- | --- | --- |
| **S2** | 0.152** | 0.200* | -0.075 | 0.078 | 0.138* | 0.230** | 0.329*** |
|  | 0.074 | 0.109 | 0.166 | 0.069 | 0.076 | 0.103 | 0.124 |
| **Gini of exporter countries** | -0.681 | -1.326* | -3.769*** | -0.454 | 0.7 | -0.666 | 0.381 |
|  | 0.478 | 0.697 | 1.311 | 0.5 | 0.555 | 0.632 | 0.817 |
| **Gini of importer countries** | 0.940** | 1.022* | -0.234 | 1.465*** | 1.535*** | 1.008* | 0.632 |
|  | 0.405 | 0.521 | 1.071 | 0.395 | 0.433 | 0.559 | 0.601 |
|  | **Wood** | **Furniture** | **Paper** | **Printing** | **Chemicals** | **Other Chem** | **Petroleum Ref.** |
| **S2** | 0.217** | 0.064 | 0.262*** | 0.220*** | 0.316*** | 0.317*** | 0.212 |
|  | 0.089 | 0.081 | 0.088 | 0.081 | 0.073 | 0.063 | 0.133 |
| **Gini of exporter countries** | -0.543 | -2.088*** | -1.269** | 0.798 | -0.37 | -2.424*** | -1.521 |
|  | 0.578 | 0.626 | 0.612 | 0.516 | 0.503 | 0.449 | 0.933 |
| **Gini of importer countries** | 1.930*** | 1.521*** | 0.383 | 0.481 | -0.237 | 0.428 | 3.170*** |
|  | 0.533 | 0.492 | 0.435 | 0.39 | 0.385 | 0.344 | 0.679 |
|  | **Petroleum and coal** | **Rubber Products** | **Plastic Products** | **Pottery** | **Glass and Products** | **Other non-met. Min.** | **Iron and Steel** |
| **S2** | 0.37 | 0.202*** | 0.329*** | 0.12 | 0.289*** | 0.357*** | 0.247** |
|  | 0.229 | 0.075 | 0.08 | 0.092 | 0.086 | 0.109 | 0.1 |
| **Gini of exporter countries** | -3.261*** | 3.353*** | -1.359** | -0.768 | -1.662*** | -0.056 | -1.297* |
|  | 1.219 | 0.586 | 0.55 | 0.647 | 0.561 | 0.642 | 0.666 |
| **Gini of importer countries** | 1.03 | 0.307 | 0.875** | 1.239** | 0.438 | 0.28 | 0.525 |
|  | 0.861 | 0.409 | 0.409 | 0.483 | 0.417 | 0.491 | 0.499 |
|  | **Non-Ferrous Metals** | **Fabric Metal** | **Machinery** | **Electrical Mach** | **Transport Equipment** | **Profession­al & Scient. Eq.** | **Other Man** |
| **S2** | -0.022 | 0.292*** | 0.156** | 0.142** | 0.209** | 0.064 | 0.143** |
|  | 0.089 | 0.062 | 0.065 | 0.069 | 0.081 | 0.064 | 0.067 |
| **Gini of exporter countries** | -0.305 | -0.425 | -0.292 | -0.336 | -0.863 | 0.37 | -0.443 |
|  | 0.656 | 0.45 | 0.462 | 0.525 | 0.616 | 0.46 | 0.438 |
| **Gini of importer countries** | -0.685 | 0.636* | 0.117 | 1.449*** | 0.633 | 0.709** | 1.589*** |
|  | 0.564 | 0.339 | 0.324 | 0.379 | 0.441 | 0.35 | 0.374 |

Note: Index S2 is described in Section 2 and measures income similarity between pairs of countries. Gini denotes the Gini inequality index. *, **, *** denote statistically significance at the 10, 5 and 1 percent level, respectively.
